# The Effect of Television and Print News Stories on the Nocebo Responding Following a Generic Medication Switch

**DOI:** 10.32872/cpe.v2i2.2623

**Published:** 2020-06-30

**Authors:** Kate MacKrill, Greg D. Gamble, Keith J. Petrie

**Affiliations:** a Department of Psychological Medicine, University of Auckland, Auckland, New Zealand; b Department of Medicine, University of Auckland, Auckland, New Zealand; Philipps-University of Marburg, Marburg, Germany

**Keywords:** nocebo effect, television media, print media, side effects, medication switch, generic medicine

## Abstract

**Background:**

Following a nationwide switch to a generic antidepressant, a series of negative media stories publicised the experiences of some patients having side effects following the switch. This occurred first in print media and five months later it occurred again in television news. In this study we examined the effect of television news stories compared to print stories on adverse drug reaction reporting. We also examined the change in reporting rate of specific side effects mentioned in the TV news bulletins.

**Method:**

Using an interrupted time series analysis of data from a national adverse reactions database, we compared the number of adverse reaction reports after the print and television coverage and the changes in reporting rate of side effects mentioned and not mentioned in TV news stories.

**Results:**

We found a significant increase in adverse reaction reports following TV news items that discussed patients’ reports of side effects following the medication switch (interruption effect = 73.25, p = .046). The reporting rate of symptoms mentioned in the TV news bulletins also increased, in particular suicidal thoughts (interruption effect = 23.60, p = .031). The effect of TV stories on adverse reaction reports was 211% greater than the print articles.

**Conclusions:**

Television stories have a much stronger effect than print media on nocebo responding and specific symptoms mentioned in the bulletins have a direct influence on the type of side effects subsequently reported. Media guidelines should be developed to reduce the negative public health effects of media coverage following medication switches.

## Abstract (highlights)

- This study shows that television news items have a considerably greater effect on the rate of adverse reaction reporting than print media.
- The specific side effects mentioned in the television coverage, especially suicidal thoughts, showed an increase in reporting.
- This study provides further evidence that media coverage of side effects can induce a nocebo effect and have negative effects on public health.

Negative media stories about medication can increase public anxiety and lead to a reduction in the use of the drug highlighted in the news story. Studies have shown that media coverage of the negative effects of statin medication is linked to the early discontinuation of the drug by patients in the United Kingdom (Matthews et al., 2016), Australia (Schaffer, Buckley, Dobbins, Banks, & Pearson, 2015) and France (Saib et al., 2013) and led to a subsequent increase in the rates of heart attacks and cardiac deaths in Denmark (Nielsen & Nordestgaard, 2016). While drops in rates of antidepressant dispensing have also been reported in the United States after widespread media coverage linking antidepressant medication to a possible increase in the risk of suicidal behaviour in young people (Yu et al., 2014).

Negative news coverage can also lead to an increase in the rate of adverse reactions reported to medication due to the nocebo effect (Petrie & Rief, 2019). This typically occurs following publicity about a particular drug’s side effects, which increases the reporting of the specific side effects mentioned in the story, due to common symptoms being misattributed to the effect of the medicine (Tan et al., 2014). An increase in the rate of adverse drug reaction reports was shown in New Zealand following television news stories reporting that patients were experiencing problems after the appearance of a common thyroid replacement medication had changed due to a shift in manufacturing plant (Faasse et al., 2009; Faasse et al., 2012). A large increase in the adverse drug reactions reports to statins was also documented following a Dutch television programme on the benefits and risks of statins (Van Hunsel et al., 2009).

It seems likely that television may have a stronger effect on the nocebo response than print media, although this has not been formally investigated. Despite the increased role of the internet and a drop in the number of young people watching, television still reaches a larger audience than other forms of news media (Gollust et al., 2019). Television news is also seen as having an important role in surveillance, by informing the public what health risks to be vigilant of and concerned about (Brosius & Kepplinger, 1990). Television news stories about health risks also typically make more use of individual case studies and individual narratives as a key part of the story, which can play an important role in social modelling of side effects (Faasse & Petrie, 2016), as well as causing an overestimation of the likelihood of a health problem occurring (Gollust et al., 2019).

A recent nationwide switch from a branded to generic antidepressant medicine in New Zealand in 2017 provided us with the opportunity to investigate the impact of newspaper stories on the nocebo effect. In this previous study we examined how newspaper stories published in February and April 2018 influenced side effect reporting up to July 2018 (MacKrill et al., 2019). We found the number of side effects, particularly those mentioned in the stories, and complaints of reduced dug efficacy increased immediately after the newspaper stories before returning to baseline levels. However, later in the year after our paper was submitted, the medicine switch received more media attention, this time from television news. Four TV news stories were broadcast from September 2 to November 30 and discussed patients’ negative reactions following the generic venlafaxine switch. The television news coverage of the same generic switch allowed us to quantify the relative impact of newspaper and television media on the nocebo response. Based on previous research we hypothesised that television news would have a larger impact. We also investigated the hypothesis that the specific side effects mentioned in the television news reports would increase adverse reaction reports to the national Centre for Adverse Reactions Monitoring (CARM), compared to previously equivalently reported side effects not mentioned in the television bulletins.

## Method

### Media Coverage

#### Newspaper Articles

In February and April 2018, two of New Zealand’s largest print media outlets published three newspaper and online articles discussing a small group of patients’ adverse reactions to the new generic version of the antidepressant venlafaxine (see Table 1). The previous year, 45,000 patients prescribed either the branded originator or a generic version of venlafaxine were switched to another generic, Enlafax. This compulsory nationwide switch had been initiated by Pharmac – the New Zealand government’s pharmaceutical agency. The articles described patients’ concerns that Enlafax was less effective and was causing side effects such as suicidal thoughts, nausea and headaches (see MacKrill et al., 2019 for further details of the newspaper reports).

**Table 1:** New Zealand Print and Television Media Coverage of the Venlafaxine Brand Change

Date	News outlet	Item title	URL
Print media			
February 28 2018	New Zealand Herald	Patients say generic Pharmac-funded version of antidepressant venlafaxine left them depressed, anxious	https://www.nzherald.co.nz/nz/news/article.cfm?c_id=1&objectid=12002918
February 28 2018	Stuff.co.nz	Anti-depressant swap: Sufferers claim generic drug is harming their condition	https://www.stuff.co.nz/national/health/101628317/antidepressant-swap-sufferers-claim-generic-drug-is-harming-their-condition
April 27 2018	Stuff.co.nz	Fight over Pharmac's switch to generic anti-depressant brand continues	https://www.stuff.co.nz/national/health/99388645/fight-over-pharmacs-switch-to-generic-antidepressant-brand-continues
Television media			
September 2 2018	One News	Growing number of patients questioning Pharmac's decision to fund a different brand of anti-depressant	https://www.tvnz.co.nz/one-news/new-zealand/growing-number-patients-questioning-pharmacs-decision-fund-different-brand-anti-depressant
September 26 2018	One News	Patients reporting life-threatening side effects from new antidepressant	https://www.tvnz.co.nz/one-news/new-zealand/patients-reporting-life-threatening-side-effects-new-antidepressant
October 20 2018	One News	Mental health specialists question new antidepressant's effectiveness	https://www.tvnz.co.nz/one-news/new-zealand/mental-health-specialists-question-new-antidepressants-effectiveness?auto=5851184169001
November 30 2018	One News	Patients claim discrimination after Medsafe warns about joint supplement but not antidepressant	https://www.tvnz.co.nz/one-news/new-zealand/patients-claim-discrimination-after-medsafe-warns-joint-supplement-but-not-antidepressant?auto=5973439901001

#### Television News Items

Five months after the print coverage, the venlafaxine brand change featured in a series of primetime news items on One News, New Zealand’s largest television news broadcaster. The first item aired on September 2 and discussed the increasing number of patients questioning Pharmac’s decision to fund a generic version of venlafaxine. Three patients were interviewed and stated that Enlafax had serious side effects, including increased suicidal ideation. While it is estimated that 2.4 of 4.8 million New Zealanders watch television each day (ThinkTV, 2018), RatingPoint, a television viewership database by analytics company Nielsen, shows that One News had an estimated audience of 679,500 viewers on September 2.

Later that month on September 26, another news item stated that more than 200 people had reported adverse reactions from the new generic some of which were considered life threatening. The side effects specifically mentioned were thoughts of self-harm and suicide, nightmares and feeling depressed. A General Practitioner was interviewed for the item and stated that the side effects were linked to Enlafax and called for patients’ previous medication to remain available as an alternative to the generic. This news bulletin received slightly fewer views at 577,100.

On October 20, One News broadcast a third item on the venlafaxine brand change. This media report included interviews with patients as well as two mental health specialists who questioned the effectiveness of the generic Enlafax. Patients reported feeling disorientated, having a foggy brain and experiencing brain zaps. In a statement, Pharmac and Medsafe (New Zealand’s medicine’s safety authority) stood by the decision to change the funded brand of venlafaxine, emphasising that the medications are pharmaceutically identical. This item received 395,000 views, the lowest of the four items.

The last media report on November 30 discussed patients’ claims of discrimination, as the New Zealand government’s Ministry of Health had released two public health warnings about an over-the-counter supplement but had not issued a warning about venlafaxine, despite patient complaints. This item received 540,600 views and no side effects were mentioned. All four One News items were aired early in the nightly news bulletin between 6pm to 6:15pm. In 2018, One News had the highest ratings of all programmes in New Zealand and was the most watched news programme (Nielsen, 2018).

### Outcome Measures

#### Number of Adverse Reaction Reports

The primary variable of interest to this study was the number of adverse reaction reports submitted to CARM each month. Both healthcare professionals and patients can submit a report describing a suspected adverse reaction from a medicine or vaccine directly to CARM. Adverse reaction data was collected from October 2017 to March 2019 which covered a four-month period before the print articles (February 28 – April 27) to four months after the TV bulletins (September 2 – November 30).

#### Total Side Effects and Decreased Therapeutic Response

The total number of side effects reported each month was calculated by summing each patient’s side effect reports excluding decreased therapeutic response, which was calculated separately.

#### Specific Side Effects

We calculated the reporting rate for suicidal thoughts, foggy brain and brain zaps that were mentioned in the television news items. The CARM side effects categories of suicidal ideation, suicidal tendency, suicidal attempt, thoughts of self-harm, and intentional self-injury were summed and recoded as suicidal thoughts. Both foggy brain and brain zaps mentioned in the television items do not have specific terms in the CARM database. We used reports of fuzzy head and electrical shock sensations as the closest coded categories. We compared the side effects that were mentioned in the television coverage with three control side effects that were not mentioned in television bulletins but were reported at similar rates prior to the media coverage. These control side effects were dizziness, drug withdrawal syndrome and irritability.

### Statistical Analyses

Three analyses were conducted to investigate the study hypotheses. An interrupted time series analysis was conducted to determine whether the television news items were associated with an increase in CARM reports, total side effects, reports of decreased therapeutic response, and the specific side effects of suicidal thoughts, foggy brain, brain zaps, dizziness, drug withdrawal syndrome and irritability. An automated integrated moving average model (ARIMA [1,0,1]) was used. To indicate the presence of the television media in the model, an independent binary variable was created with the months September to December 2018 coded 1 and the five baseline pre-media months coded 0. This analysis produces an estimated interruption effect (the change in rate between the months coded 0 and 1) and indicates whether this is a significant change.

In addition to this analysis, the number of adverse reaction reports was modelled using general linear modelling (GLIMMIX) assuming a Poisson distribution to test for differences in the total number of reports in discrete time periods: 5 pre-media baseline months, 3 months during the print media stories, the next 3 months (a pre-TV, no media period), 4 months during which television media reports appeared, and an additional post-TV 3 months. These time periods were pragmatically determined: initiated by the start of each type of media report and ending when reports had returned to the pre-media reporting baseline. Tukey’s HSD test was used to protect the overall 5% significance level after pairwise post hoc comparison of time periods.

To examine the effect of print versus television media on adverse reporting, a Poisson Events Test was conducted comparing total number of reports between pairs of months, specifically the peak month of reporting during the print media period and the peak during the TV media period. Percentage change was used to describe the effect of the print and television media on number of CARM reports. Analyses were conducted in SAS (v9.4 SAS Institute Inc., Cary, NC) and alpha level of .05 was considered significant for all analyses.

## Results

### Number of Adverse Reaction Reports

From August 2018 to March 2019 there were 341 adverse reaction reports to CARM, with 317 of these occurring during the four-month period when the television items aired. The average age of reporters was 44.3 years old and 79.1% were female. These demographic proportions are similar to the total population of people taking venlafaxine in New Zealand, the median age range being 40-49 years and 64.5% identifying as female (MacKrill & Petrie, 2018).

**Figure 1:**
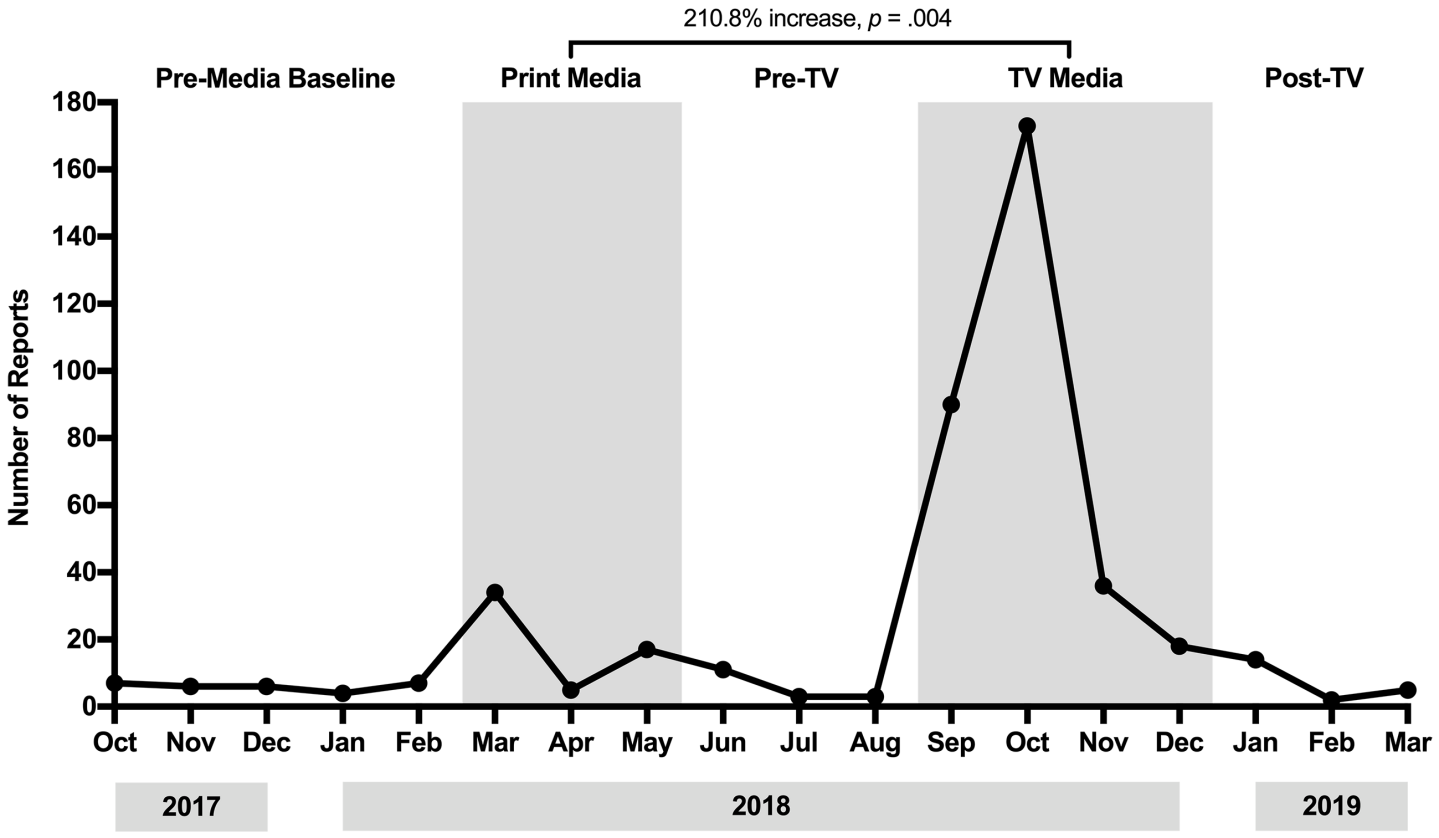
The Effect of Television Compared to Print Media Stories on Total Number of Adverse Reaction Reports to the Centre for Adverse Reactions Monitoring Following a Switch to Generic Venlafaxine *Note.* The number of adverse reaction reports during pre-media baseline was not significantly different to the reporting rate pre (GLIMMIX *p* = .220) or post (GLIMMIX *p* = .120) the television coverage.

The first aim of this study was to examine the impact of the television coverage on adverse event reporting and compare this with what was observed following the print media. There were significant differences between time periods in the number of adverse reaction reports (GLIMMIX *p* < .001). In the five months before any print or television media (October 2017 to February 2018), there was an average of 6.00 (*SD* = 1.23) adverse reaction reports to CARM per month. However, in the four months where the television coverage occurred, CARM reports significantly increased to an average of 79.25 (*SD* = 60.26) reports per month (interruption effect [IE] = 73.25, *p* = .046). Comparing the average effect of print versus television media, CARM reports following the television coverage were 210.8% greater than those that followed the print (mean number of reports = 25.50, *SD* = 12.02), which was a significant difference (GLIMMIX *p* = .004) as shown in Figure 1. A Poisson Events Test showed that the peak month of adverse reaction reporting during the television coverage was 408.8% greater than the peak during the print media period (*p* < .001).

### Total Number of Side Effects and Decreased Therapeutic Response Reports

Individual CARM reports submitted from August 2018 to March 2019 listed an average of 2.88 side effects attributed to Enlafax. The rate of side effect reporting significantly changed from baseline to post-television. The total number of side effects reported to CARM significantly increased from an average of 7.00 reports (*SD* = 4.18) per month before any media coverage to 235.75 (*SD* = 184.77) following the television items (IE = 228.75, *p* = .042). Reports of ‘decreased therapeutic response’ increased from 4.00 (*SD* = 2.12) before the media to 52.25 (*SD* = 39.35) after the television, however this was not a statistically significant change (*p =* .064).

### Specific Side Effects Reports

**Figure 2:**
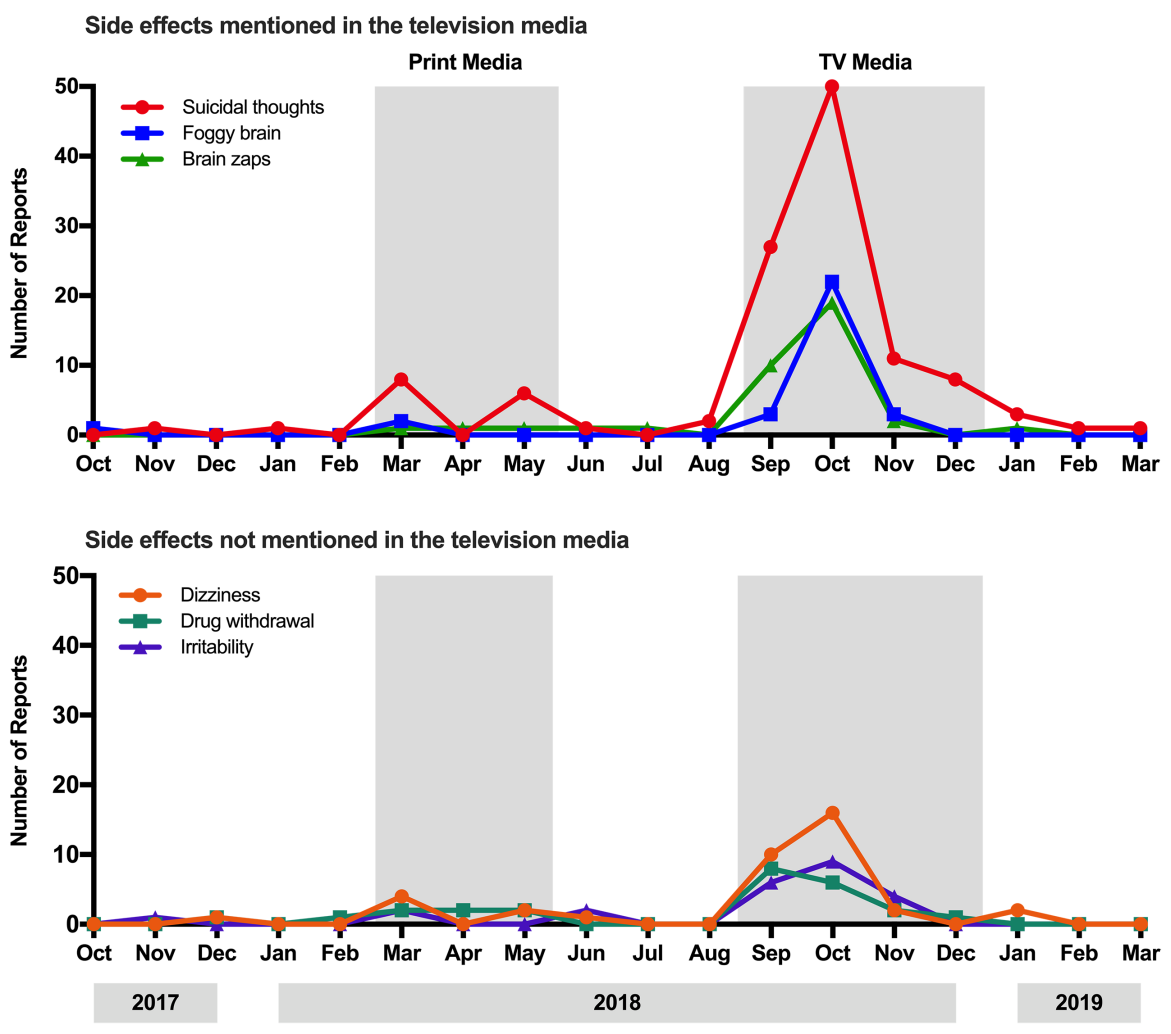
Number of Reports of Side Effects Mentioned in the Television News Reports on the Venlafaxine Switch Compared to Side Effects That Were not Mentioned

We investigated the change in reporting of rate of three side effects that were mentioned in the television coverage (Figure 2). The generalised mixed model and interrupted time series analyses both showed a significant increase in reports of suicidal thoughts from an average of 0.40 (*SD* = 0.55) in the five months before any media coverage to 24.00 (*SD* = 16.66) following the television (GLIMMIX *p* = .029; IE = 23.60, *p* = .031). The reporting rate of foggy brain did not show a statistically significant increase in the number of reports in each time period (GLIMMIX *p* = .160; IE = 8.13, *p* = .160), however there was an increasing trend with reports going from 0.20 (*SD* = 0.40) before the media coverage to 8.33 (*SD* = 9.74) after the television item aired in October. While there were no reports of brain zaps during the pre-media period, the rate increased to an average of 7.00 (*SD* = 8.52) but this was not significantly different to baseline (GLIMMIX *p* = .150; IE = 7.00, *p* = .098).

Finally, we examined whether there was a change in the reporting rate of three side effects that were not mentioned in the media (Figure 2). Reports of dizziness increased from an average of 0.20 (*SD* = 0.40) over the five baseline months to 7.00 (*SD* = 6.40) following the television coverage, which has a statistically significant interruption effect (IE = 6.80, *p* = .024), however, the general linear model was not statistically significant (GLIMMIX *p* = .110). The reporting rate of drug withdrawal syndrome did not significantly change from 0.40 (*SD* = 0.49) to 4.25 (*SD* = 2.86) after the television media (GLIMMIX *p* = .470; IE = 3.85, *p* = .180). Similarly, reports of irritability did not significantly change from 0.20 (*SD* = 0.40) to 4.75 (*SD* = 3.27) (GLIMMIX *p* = .011; IE = 4.55, *p* = .240).

## Discussion

### Main Findings

This study found a significant increase in the number of adverse reaction reports following a switch to a generic formulation of venlafaxine, which corresponded to the broadcast of four television news items that discussed this medication change. In line with our hypothesis, we found the effect of TV stories on adverse reaction reports to CARM was significantly higher than print media, causing an approximately 200% greater rise in adverse reaction reports than the publication of the print articles earlier that year. Television news also had a 400% greater peak in reported adverse reports compared to print media, indicating a much stronger nocebo response. There was also partial support for the hypothesis that the specific symptoms mentioned in the TV coverage would be reflected in subsequent side effect reporting. There was an increase in the reporting of side effects mentioned in the television items, especially suicidal thoughts, and although this was generally larger than the symptoms that were not mentioned, it could be that TV coverage causes a greater awareness of side effects in general, rather than being restricted to those specifically mentioned in the bulletins.

Looking at the reasons why TV has a much stronger effect than print media, it seems unlikely that this is due to the use of expert opinion or difference in the amount of coverage (3 print versus 4 TV stories). A more likely explanation is that television contains a stronger and more impactful modelling element by including real patient stories and experiences that can be easily identified with by viewers (Faasse, Grey, Jordan, Garland, & Petrie, 2015; Faasse & Petrie, 2016).

### Comparison With Other Studies

The results are consistent with data in the medical area showing intense negative media coverage on statins was followed by an increase in patients stopping the drug (Matthews et al., 2016; Schaffer et al., 2015). The results also align with previous work on TV news stories. For example, the Thyroxine drug scare produced an increase in both symptom reporting and the specific symptoms mentioned in bulletins, increasing adverse reaction reports by 1,866% following the first television news story (Faasse et al., 2012). The current study showed an even larger increase in adverse reaction reports after the first television news bulletin of 4,283%. More widely, the data are consistent with the powerful social modelling effects of TV in the context of suicidal behaviour (Hawton & Williams, 2002), mass shootings (Meindl & Ivy, 2017) and the transmission of acute stress following terrorist attacks (Holman et al., 2014). The unique contribution of this paper is to quantify the relative impact of television compared to print media and to demonstrate how much more impact TV has in the context of a health scare.

It may be that the nature of the population taking venlafaxine could have influenced the strength of the nocebo response. The indications for the drug are for depressive and anxiety disorders and the nocebo effect has been shown to occur more frequently in patients being treated for psychological conditions (Weissenfeld et al., 2010). Individuals taking venlafaxine may have been more reactive to negative stories, increasing their overall concerns about the medication. It is likely that the increased nocebo response apparent following media coverage arose from an overall increase in anxiety, increased expectations of side effects and greater personal monitoring of the side effects specifically mentioned in these bulletins (Crichton et al., 2014; Faasse & Petrie, 2016; Petrie, Moss-Morris, Grey, & Shaw, 2004; Petrie & Rief, 2019). Of particular concern in such situations is the media transmission of suicidal thoughts, which seem to be easily converted into increased rates of suicidal ideation following both print and television media stories and possibly greater rates of suicidal behaviour, although this has yet to be determined in this situation.

### Strengths and Limitations

The study is limited by reliance on reporting to the national centre and is likely to be a low estimate of the true rate of nocebo response following the media stories as many patients would not have reported symptoms to CARM or to a health professional. It is also likely that many doctors may not have taken the time to file a report. Previous studies estimate that reports to a national database are less than 10% of adverse drug reactions (McGettigan et al., 1997). As the reports to CARM are de-identified we are unable to examine other personal characteristics that may be associated with increased or decreased nocebo responding. However, people who are older, female and with lower medicine efficacy beliefs have been shown to report more side effects following a generic medicine switch (MacKrill & Petrie, 2018). It should be also noted that the current study only had access to adverse reaction reports per month. This makes it more difficult to separate out media effects from background noise compared to a finer grain of measurement such as weekly reports.

In conclusion, we believe this is the first study to compare the effect of both print and television media on medication adverse event reporting. We found television news stories have around a 200% stronger effect on nocebo responding than print media and cause an immediate increase in overall adverse reaction responding as well as influencing the type of symptoms reported following the coverage. Television news coverage can easily increase overall anxiety about a medication and cause individuals to focus on their symptoms as possible side effects. The transmission of symptoms of suicidal ideation is of special concern as there is good evidence of a strong modelling effect on suicidal behaviour from media stories (Hawton & Williams, 2002). We believe the data indicate that media guidelines should be developed to reduce the possible harm from stories that focus on dramatic negative effects reported by individual patients to include information from a wider range of professionals and agencies as well as including information about the nocebo response.
